# PERCEPTION AND WILLINGNESS TO THE UPTAKE OF COVID-19 VACCINE AMONG HOUSEHOLD-HEADS IN A RURAL COMMUNITY OF SOUTH-WESTERN NIGERIA

**DOI:** 10.21010/Ajidv17i2.1

**Published:** 2023-03-29

**Authors:** ADEWOYE Kayode Rasaq, OLALUBI Oluwasogo A, AREMU Shuaib Kayode, ALAO Taiye Adeyanju, EKPO David Sylvanus, IPINNIMO Tope Michael, ADENIYI Makinde Adebayo, IBRAHIM Azeez Oyemomi, SANNI Taofeek Adedayo, ACHEBE Chijioke Cosmas, BAKARE Adewumi, OREWOLE Tesleem Olayinka, ABIOYE Opeyemi Oladipupo

**Affiliations:** 1 Department of Community Medicine, Afe Babalola University, Ado-Ekiti, Ekiti State, Nigeria; 2 Department of Community Medicine, Federal Teaching Hospital, Ido-Ekiti, Ekiti State, Nigeria; 3 Department of Public Health, Kwara State University, Molete, Kwara State, Nigeria; 4 Department of Otorhinolaryngology, Afe Babalola University, Ado-Ekiti, Ekiti State, Nigeria; 5 Department of Community Medicine and Primary Health-Care, Federal Medical Centre, Abeokuta, Ogun State, Nigeria; 6 Department of Family Medicine, Federal Teaching Hospital, Ido-Ekiti, Ekiti State, Nigeria; 7 Department of Radiology, Federal Teaching Hospital, Ido-Ekiti, Ekiti State, Nigeria; 8 Department of Obstetrics and Gynaecology, Federal Teaching Hospital, Ido-Ekiti, Ekiti State, Nigeria; 9 Department of Anaesthesia, Afe Babalola University, Ado-Ekiti / Federal Teaching Hospital, Ido Ekiti, Ekiti State, Nigeria

**Keywords:** Perception, Willingness, Uptake, COVID-19 vaccine, Household heads, Nigeria

## Abstract

**Background:**

The COVID-19 pandemic and its vaccine have been met with varying perceptions that may have both negative and positive effects on the willingness to uptake the COVID-19 vaccine. The study is set to determine the perception and willingness of the household heads to the uptake of COVID-19 vaccine in a rural community in Southwestern, Nigeria.

**Materials and Methods:**

A cross-sectional study was carried out among 409 household heads selected through a multistage sampling technique. The instrument of data collection was a semi-structured interviewer-administered questionnaire using the Health Belief model constructs. Data were analyzed with IBM SPSS version 21.0 and Pearson’s Chi-square test was used to determine the association between perception and willingness to uptake vaccine. P<0.05 was taken as significant at 95% confidence interval.

**Results:**

The majority of the unvaccinated respondents in the study were not willing to take the COVID-19 vaccine (60.1%). There was a poor perception of the susceptibility/severity of unvaccinated respondents to COVID-19 infection and a poor perception of the benefit/barrier to the uptake of the COVID-19 vaccine. Perception of susceptibility and severity of COVID-19 infection were statistically related to the willingness to uptake the COVID-19 vaccine.

**Conclusion:**

There should be an increase in awareness campaigns to change the perception of people positively to COVID-19 infection and uptake of the COVID-19 vaccine.

## Introduction

Coronavirus disease 2019 (COVID-19) has led to more than 6 million deaths and has infected over 600 million people worldwide. The pandemic has disrupted human life at both local and international levels, thus necessitating its declaration as a public health emergency of international concern by the World Health Organization (WHO, 2020). Non-availability of the highly efficacious antivirus drug and the virulence of the virus has further heightened the global concern about the disease. The effort has been geared towards the primary prevention of the disease via health promotion and the development of vaccines to build herd immunity. The vaccine was discovered to be the most cost-effective in controlling the COVID-19 pandemic. Scientists around the world have developed vaccines against the virus. These are Ribonucleic acid **(**RNA) vaccines from (Pfizer-BioNTech and Moderna), a conventional inactivated vaccine from Sinipharm, Bharal Biotech and viral vector vaccines from Gamalaya Research Institute and Oxford Astrazeneca. They are being used in recent vaccination programs of many countries including the United States, United Kingdom, China, Russia, India, and Nigeria (Kim *et al.*, 2021). The WHO identified vaccine hesitancy as one of the top primary global health threats. It defined vaccine hesitancy as the reluctance and refusal to vaccinate despite vaccine availability (Jacob *et al.*, 2015). Several studies indicate that vaccine hesitancy, acceptance, and refusal would be the biggest challenge for many countries in achieving desired vaccination coverage (>70%), especially for COVID-19 vaccination (Contanssa *et al.*, 2021). The COVID-19 pandemic has been met with varying perceptions ranging from negative to positive perceptions. The overabundance of information about the disease which comprises both right and wrong information has contributed to varying perceptions in the community which may have either positive or negative effects on the willingness to uptake the COVID-19 vaccine. A nationwide poll conducted in the United States and a cross-sectional study done in China showed a higher rate of COVID-19 hesitancy among rural residents (Lazarus *et al.*, 2021). Acceptance of a COVID-19 vaccine and willingness to partake depend on perception of susceptibility to COVID-19 infection, belief about the severity of COVID-19 infection, perceived benefit of the vaccine in preventing COVID-19 infection, perceived barriers, presence of chronic diseases and knowing someone in the community who has had COVID-19 infection. A study in Osun State, Nigeria showed that 59.1% of the respondents were willing to take the COVID-19 vaccine but the number declined sharply if they were asked to pay for it (Akinyemi *et al.*, 2021). However, there is a paucity of information and a dearth of empirical evidence in terms of the availability of data on vaccine hesitancy, perception, acceptance, and uptake of the COVID-19 vaccine in rural areas of Nigeria.

Exploring significant Health Belief Model constructs that influenced the uptake of COVID-19 vaccination may be crucial for tailored interventions to enhance the acceptance of the vaccines. Data on vaccine acceptance in low- and middle-income countries like Nigeria is limited. A limited study has been carried out in Nigeria on COVID-19 vaccine acceptance and uptake. Most of the studies done in Nigeria were in the urban area but this study will focus on the rural community. The study will offer valuable baseline information to assess the willingness to vaccinate in the context of the COVID-19 pandemic. The study will provide information to policy makers to formulate policy on ways to improve the acceptance and uptake of the COVID-19 vaccine among the populace. This research set out to determine the perception of unvaccinated participants to COVID-19 infection and COVID-19 vaccine using the Health Belief model constructs, determine the uptake of the COVID-19 vaccine by the study participants, the willingness of the unvaccinated respondents or participants to take COVID-19 vaccine and to study the relationship between perception/sociodemographic variables and willingness to uptake COVID-19 vaccine.

## Materials and Methods

The study design was a descriptive cross-sectional design that employed a quantitative method of data collection. Quantitative data was collected through the use of pre-tested semi-structured, paper-based, interviewer-administered questionnaires to assess perception and willingness to uptake the COVID-19 vaccine. The study populations were all household heads that consented to participate in the study and residents of the Ido-Ekiti community. Representative of the household head are included in the absence of the household head. Excluding from the study are subjects with chronic illness and mental illnesses.

The study was conducted over one month period (July 2021).

Fisher’s formula for a population >10,000 was used to determine the sample size for this study.


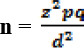


Where n= desired sample size

z = standard normal deviate, 1.96

p = proportion of willing to uptake of covid 19 vaccine = 0.59

q = 1 – p (1-0.59=0.41)

d = degree of accuracy desired = 0.05


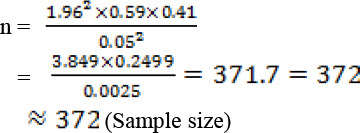


The non-response sample size was calculated as 10% of the total sample size= 37.2.

37.2+372=409.2 == 409 (sample size)

### Sampling Technique

The respondents were selected using a multistage sampling technique

Stage 1- There are 2 political wards in the Ido-Ekiti community. One of them was selected using simple random sampling by balloting.

Stage 2- From the number of settlements under the political ward picked in stage 1, 4 settlements were picked using simple random sampling by balloting

Stage 3- The house numbering exercise was carried out in all the 4 settlements picked in stage 2 followed by the generation of household listings for all the four settlements picked in stage 2. The household list generated served as the sampling frame.

Stage 4- Systematic sampling was used to select 409 households for the study using the household listing generated in stage 3 as a sampling frame

Stage 5- The household head in each of the households selected in stage 4 was interviewed using interviewer administered semi-structured questionnaire

### Inclusion and Exclusion Criteria

Representatives of the household head are included in the absence of the household head. Excluding from the study are subjects with chronic illness and mental illnesses.

### Research Instrument

The instrument that was used for data collection is a semi-structured, interviewer-administered questionnaire which was adapted from previous work and was validated. The questionnaire was written in English language and the contents were interpreted by the researcher and research assistants to those participants who do not understand the English language.

The validity and reliability of the instrument were guaranteed by the pretest of the questionnaires on 41 subjects in Otun-Ekiti (a rural community situated about 20 kilometres from the study area). Appropriate corrections were made to the questionnaire after pre-testing where necessary.

### Procedure for data collection

Three research assistants were recruited and trained, and the contents of the questionnaire were thoroughly explained and interpreted by the research assistants during their training. They assisted in the data collection and the interpretation of the content of the questionnaire to non-English speaking respondents. The research assistant interviewed the household heads in any comfortable space within their house after their consent must have been obtained.

### Data Analysis

Data were collected, checked for errors, and entered manually into the computer for statistical analysis. IBM SPSS version 21.0 was used for the data analysis. Data analysis was done using descriptive and inferential techniques. Descriptive statistics of the data were presented in frequencies, percentages, means, and standard deviation, using tables. The Inferential procedure involved the use of Pearson’s Chi-square that was used to check if there is an association between variables and to ascertain whether the association is significant (p<0.05) or not.

### Ethical Considerations

Following the principles governing research involving human participants, the researchers undertook the following steps to uphold respondents’ ethical rights: All participants were required to give informed consent before participating in the study. Those who were unwilling to participate were excluded from the study. The confidentiality of their response and their anonymity was fully guaranteed. The purpose and benefits of the research were clearly explained to the respondents before the questionnaires were administered. Respondents received a detailed description of the study, confidentiality provisions, and the fact that their participation is voluntary and they could withdraw at any point if they so wish. The principles of autonomy and confidentiality were upheld.

The study approval (Protocol number: ERC/2021/06/12/570A) was given by the research and ethical committee of the Federal Teaching Hospital, Ido-Ekiti.

## Results

Table 1 shows the sociodemographic variables of the respondents. The mean age ± standard deviation of the respondents was 42.2 ± 12 years. More than two thirds of the respondents are of the Yoruba ethnic group (68.2%). More than half of the respondents (59.9%) had tertiary education and are Christians (62.1%). Close to half of the respondents interviewed (42.8%) are civil servants and learn between N10,000 – N50,000 per month (40.6%).

**Table 1:** Socio-demographic characteristics of respondents.

Variable	Frequency N = 409	Percentage (%)
**Age group**		
Young adult (18 to 35 years)	153	37.4
Middle age (36 to 55 years)	187	45.7
Older adult (above 55 years)	69	16.9
*Mean age ± SD*	*42.2 ± 12.0*	
**Sex**		
Male	182	44.5
Female	227	55.5
**Ethnicity**		
Yoruba	279	68.2
Others	130	31.8
**Education**		
None	26	6.4
Primary	38	9.3
Secondary	100	24.4
Tertiary	245	59.9
**Religion**		
Islam	108	26.4
Christianity	254	62.1
Others	47	11.5
**Religion**		
Civil servant	175	42.8
Retiree	61	14.9
Trader/ self-employed	148	36.2
Unemployed	25	6.1
**Income (in Naira)**		
<10,000	73	17.8
10,000 - <50,000	166	40.6
50,000 - <100,000	117	28.6
≥100,000	53	13.0

In Table 2, the majority of the respondents had not been vaccinated against COVID-19 (88.8%). More than half of the respondents (60.1%) that had not been vaccinated against COVID-19 indicated their non-willingness to the vaccine. The major reason for non-willingness to take the COVID-19 vaccine among the respondents that had not been vaccinated is distrust in the government and health organizations (72.5%). More of the respondents that had not been vaccinated (90.6%) indicated their non-willingness to take the COVID-19 vaccine if the vaccination is not free.

**Table 2:** Uptake and willingness to uptake the COVID-19 vaccine.

Variable	Frequency N = 409	Percentage (%)
**Ever vaccinated against COVID-19**		
Yes	46	11.2
No	363	88.8
**Willingness to take COVID -19 vaccine (n = 363)**		
Yes	145	39.9
No	218	60.1
Reason (s) for NOT willing (n = 218)*		
Fear of side effects	119	54.6
Efficacy concern	87	39.9
Distrust in government and health organizations	158	72.5
It is not just necessary	58	26.6
**Willingness, if vaccination is not FREE (n = 363)**		
Yes	34	9.4
No	329	90.6
**Reason for non uptake COVID-19 vaccine (n = 363)**		
The COVID-19 vaccine is not available or does not reach my ward	345	95.0
Was asked to come back because the certificate or card was not available	18	5.0

* *Multiple responses*

Table 3 shows the perception to uptake COVID-19 vaccine among un-vaccinated respondents. On the unvaccinated respondent’s perception of susceptibility to COVID-19 infection, more than half (55.6%) agreed they are worried about the likelihood of getting COVID-19 infection.

**Table 3:** Perception to uptake COVID-19 vaccine among un-vaccinated respondents (n = 363).

Perception	Agree n (%)	Disagree n (%)
**Susceptibility**		
My chance of getting the COVID-19 vaccine is great in the next few months	55 (15.2)	308 (84.8)
I am worried about the likelihood of getting COVID-19	202 (55.6)	161 (44.4)
Getting COVID-19 is currently a possibility for me	88 (24.2)	275 (75.8)
I am worried about COVID-19 because of my advancing age	70 (19.3)	293 (80.7)
I am concerned about COVID because of my underlining concomitant chronic health problem	157 (43.3)	206 (56.7)
**Severity**		
The complication of COVID-19 is serious	265 (73.0)	98 (27.0)
I will be very sick if I get COVID-19	175 (48.2)	188 (51.8)
**Benefit**		
Vaccination is a good idea because it makes me less worried about COVID-19	177 (48.8)	186 (51.2)
Vaccination decreases my chance of getting COVID-19	182 (50.1)	181 (49.9)
**Barrier**		
I am worried that the side effects of the COVID-19 vaccine would interfere with my activities	157 (43.3)	206 (56.7)
I am scared of trauma, the pain of injection	169 (46.6)	194 (53.4)
I am concerned about the efficacy of the COVID-19 vaccine	202 (55.6)	161 (44.4)
Reports and observations from close associates previously vaccinated discouraged me	194 (53.4)	169 (46.6)
I am more concerned about the safety of the COVID-19 vaccine	220 (60.6)	143 (39.4)
I am more concerned about the affordability of getting COVID-19	115 (31.7)	248 (68.3)
I am more concerned that acceptance of the COVID-19 vaccine is against my religion	58 (16.0)	305 (84.0)
**Cue to action**		
I will only take the COVID-19 vaccine if I can be given more adequate information about it	274 (75.5)	89 (24.5)
I will only take COVID-19 if the vaccine is taken by many or a larger percentage of the public	205 (56.5)	158 (43.5)

Table 4 presents information on the perception of the unvaccinated respondents regarding the uptake of the COVID-19 vaccine by the scoring system. More than half of the unvaccinated respondents have a poor perception of the severity (58.7%) to covid 19 infection, benefit (54.7%), and barrier (61.2%) to the uptake of the COVID-19 vaccine. More than two-thirds of the unvaccinated respondents have poor perceptions of susceptibility to COVID-19 infection (81.3%). Less than half of the unvaccinated respondents (47.9%) have a poor perception of the cue to action to uptake the COVID-19 vaccine. More than two-thirds of the respondents that have not received the COVID-19 vaccine (69.1%) have a poor general perception of uptake COVID-19 vaccine.

**Table 4:** Assessments of respondents’ perception of uptake COVID-19 vaccine

Variable	Frequency N = 363	Percentage (%)
**Perception of susceptibility**		
Good (3 – 5)	68	18.7
Poor (0 – 2)	295	81.3
**Perception of severity**		
Good (2)	150	41.3
Poor (0 – 1)	213	58.7
**Perception of benefit**		
Good (2)	166	45.7
Poor (0 – 1)	197	54.3
**Perception of barrier**		
Good (5 – 7)	141	38.8
Poor (0 – 4)	222	61.2
**Perception of cue to action**		
Good (2)	189	52.1
Poor (0 – 1)	174	47.9
**General perception**		
Good (10 – 18)	112	30.9
Poor (0 – 9)	251	69.1

In Table 5, age (p<0.001), education (p=0.030), occupation (p<0.001), and income (p<0.001) were sociodemographic and economic factors that are statistically related to the willingness to uptake the COVID-19 vaccine among the unvaccinated respondents.

**Table 5:** Socio-demographic associates of willingness to the uptake of the COVID-19 vaccine among the unvaccinated respondents

Variable	Willingness to uptake the COVID-19 vaccine		Chi-square	p-value

	Yes n (%)	No n (%)		
**Age group**				
Young adult (18 to 35 years)	31 (25.0)	93 (75.0)	18.930	**<0.001**
Middle age (36 to 55 years)	60 (37.5)	100 (62.5)		
Older adult (above 55 years)	48 (60.0)	31 (40.0)		
**Sex**				
Male	77 (48.1)	83 (51.9)	7.981	**0.005**
Female	68 (33.5)	135 (66.5)		
**Ethnicity**				
Yoruba	92 (38.2)	149 (61.8)	0.937	0.333
Others	53 (43.4)	69 (56.9)		
**Education**				
None	4 (22.2)	14 (77.8)	8.936	**0.030**
Primary	9 (26.5)	25 (73.5)		
Secondary	32 (34.8)	60 (65.2)		
Tertiary	100 (45.7)	119 (54.3)		
**Religion**				
Islam	45 (48.9)	47 (51.1)	4.134	0.127
Christianity	84 (36.8)	144 (63.2)		
Others	16 (37.2)	27 (62.8)		
**Occupation**				
Civil servant	64 (42.1)	88 (57.9)	19.234	**<0.001**
Retiree	34 (63.0)	20 (37.0)		
Trader/ self-employed	38 (28.8)	94 (71.2)		
Unemployed	9 (36.0)	16 (64.0)		
**Income (in Naira)**				
<10,000	18 (27.7)	47 (72.3)	31.994	**<0.001**
10,000 - <50,000	49 (32.5)	102 (67.5)		
50,000 - <100,000	41 (42.3)	56 (57.7)		
≥100,000	37 (74.0)	13 (26.0)		

Table 6 shows that unvaccinated respondents’ perception of susceptibility to COVID-19 infection is statistically related to the willingness to uptake the COVID-19 vaccine (p=0.003). About 55% of the unvaccinated respondents indicated their willingness to uptake the COVID-19 vaccine as compared to 36.3% of those with poor perceptions. Respondent’s perception of the severity of COVID-19 infection is statistically related to the willingness to uptake the COVID-19 vaccine (p<0.001). Perception of barrier and cue to action were statistically related to the willingness to uptake the COVID-19 vaccine (p<0.001, <0.001) respectively. The majority of the respondents with good general perception (83%) indicated their willingness to uptake the COVID-19 vaccine and this was statistically related (p<0.001).

**Table 6:** Associations between perceptions of unvaccinated respondents about COVID-19 vaccines/infections and their willingness to uptake the vaccine(n-363)

Variable	Willingness to uptake the COVID-19 vaccine		Chi-square	p-value

	Yes n (%)	No n (%)		
**Perception of susceptibility**				
Good (3 – 5)	38 (55.9)	30 (44.1)	8.860	**0.003**
Poor (0 – 2)	107 (36.3)	188 (63.7)		
**Perception of severity**				
Good (2)	102 (68.0)	48 (32.0)	83.875	**<0.001**
Poor (0 – 1)	43 (20.2)	170 (79.8)		
**Perception of benefit**				
Good (2)	71 (42.8)	95 (57.3)	1.018	0.313
Poor (0 – 1)	74 (37.6)	123 (62.4)		
**Perception of barrier**				
Good (5 – 7)	88 (62.4)	53 (37.6)	48.510	**<0.001**
Poor (0 – 4)	57 (25.7)	165 (74.3)		
**Perception of cue to action**				
Good (2)	59 (31.2)	130 (68.8)	12.521	**<0.001**
Poor (0 – 1)	86 (49.4)	88 (50.6)		
**General perception**				
Good (10 – 18)	93 (83.0)	19 (17.0)	125.374	**<0.001**
Poor (0 – 9)	52 (20.7)	199 (79.3)		

## Discussion

Majority of the respondents had not been vaccinated against COVID-19 infection. This is expected because as of the time of collecting the data, only one million doses of the COVID-19 vaccines were available for the country. The main target of those to be vaccinated with the available vaccines were those at high risk of contracting the disease mainly health workers and people with an underline pre-morbid condition like diabetes, cancer, Human immunodeficiency virus (HIV), and other immunosuppressive disorders.

Majority of the respondents that had not been vaccinated against COVID-19 infection were unwilling to uptake the vaccine (60.1%). When the unvaccinated respondents were asked if they will be willing to uptake the vaccine if they were asked to pay some amount of money for the vaccine, most of the unvaccinated respondents (90%) indicated their unwillingness.

This finding is in tandem with what was obtained in a similar study in Osun State, Nigeria where 59.1% of the respondents were unwilling to uptake COVID-19 vaccines (Akinyemi *et al.*, 2021). In a similar study carried out in the United States among New Yorkers who had not received any dose of the vaccine, 68% of them indicated their intention to be vaccinated (New York Health, 2021).

Majority of the unvaccinated respondents have a poor perception of susceptibility and severity of COVID-19 infection and a poor perception of the benefit and barriers to the uptake of the COVID-19 vaccine. More than two-thirds of the respondents have a poor general perception of COVID-19 infection and uptake of the COVID-19 vaccine. This finding is in contrast to a finding obtained in the middle-east where the majority of the respondents interviewed had a high perception of susceptibility, severity, benefit, and self-efficacy of COVID-19 infection but lower perceived barriers and fatalistic beliefs (Elgzar *et al.*, 2020). Another study in Ibadan, Western Nigeria, showed that 79.5% of the respondents interviewed showed positive perception regarding COVID-19 vaccine usage when it is available but there is low perceived susceptibility to COVID-19 infection (Ilesanmi *et al.*, 2021). Having a high or good perception of susceptibility, and severity to COVID-19 infection and the benefit of the COVID-19 vaccine will increase the rate of adherence to preventive behaviors against COVID-19 such as the uptake of COVID-19 vaccine and all other preventive measures such as wearing a face mask.

There was a statistically related association between all health belief model constructs (susceptibility, severity, barriers, cue to action) and willingness to uptake the COVID-19 vaccine except for that of the benefit which is not related statistically to the willingness to uptake the COVID-19 vaccine. The general perception of the respondents was also found to be statistically related to the willingness to uptake COVID-19 vaccines. These findings are in contrast with the result obtained from a similar study in Iran where perceived benefit, barrier, and self-efficacy were predictive of preventive behaviors but the perception of susceptibility and severity were not significant. Another study in Osun State, Nigeria reported perception of COVID-19 and its vaccine were important factors affecting the willingness to uptake COVID-19 vaccines. The study reported a positive significant association between all constructs of perception based on the health belief model and the willingness to take COVID-19 vaccines except for perceived barriers. The Ibadan study reported that perceived susceptibility to contracting COVID-19 infection and practices to prevent COVID-19 though significant, had a weak correlation (Ilesanmi *et al.*, 2021). Also, Shahnazi *et al*. reported that perceived susceptibility and severity had no significant relationship among respondents in predicting their preventive behaviors against COVID-19 infection. However, the perceived threat construct was an important variable to predict their preventive behaviors against COVID-19 infection.

The following socio-demographic factors namely age, sex, education, and occupation were statistically related to the willingness to uptake the COVID-19 vaccine among the unvaccinated respondents (p<0.05). A similar study carried out in China revealed that people between 46-59 years of age, town dwellers who had good knowledge about COVID-19 and belief in the efficacy of the COVID-19 vaccine in preventing the infection were significantly willing to accept COVID-19 vaccination (Wan *et al.*, 2021). A population-based study in Nigeria identified increasing age, and male gender as some of the factors that influence the acceptance of COVID-19 vaccine (Tobin *et al.*, 2021). Another study in Osun State, Nigeria recognized having higher education and being a health worker as some of the factors that affect willingness to accept the COVID-19 vaccine (Akinyemi *et al.*, 2021).

## Conclusions

Willingness to uptake COVID-19 vaccines among the unvaccinated respondents was low. The majority of the respondents have a poor perception of COVID-19 infections and its vaccine using the health belief model constructs. Most of the respondents have poor general perception of COVID-19 infections and its vaccine. Perception of susceptibility and severity of COVID-19 disease is statistically related to the willingness to uptake COVID-19 vaccines among the unvaccinated respondents. Perception of barrier and cue to action to uptake COVID-19 vaccines is statistically related to the willingness to uptake the COVID-19 vaccine among the unvaccinated respondents. The general perception is also statistically related to the willingness to uptake COVID-19 vaccines among the unvaccinated respondents. Age, sex, income, and educational status are important socio-demographic variables that are statistically related to the willingness to uptake COVID-19 vaccines among the unvaccinated respondents.

Based on these findings, we recommend that the government should make the COVID-19 vaccine more readily available, accessible, and affordable to all the citizens of this country at no cost. Increase awareness campaigns by the three levels of government on COVID-19 infection, the benefit of the COVID-19 vaccine, and disabuse the mind of the people on the presumed barriers to vaccination. Every community should take it as their responsibility to protect the community members against COVID-19 by creating awareness and sensitization campaigns for its members.

### Conflicts of Interest

The authors declare that there is no conflict of interest associated with this study.

**List of Abbreviations:**
(COVID-19); Coronavirus disease 2019
(HIV); Human immunodeficiency virus
(RNA); Ribonucleic acid
(WHO).: World Health Organization
